# Application of Response Surface Methodology (RSM) for the Optimization of Chromium(III) Synergistic Extraction by Supported Liquid Membrane

**DOI:** 10.3390/membranes11110854

**Published:** 2021-11-04

**Authors:** Jakub Rajewski, Agnieszka Dobrzyńska-Inger

**Affiliations:** ŁUKASIEWICZ Research Network-New Chemical Syntheses Institute, Al. Tysiąclecia Państwa Polskiego 13a, 24-110 Puławy, Poland; agnieszka.dobrzynska-inger@ins.lukasiewicz.gov.pl

**Keywords:** response surface methodology, supported liquid membrane, chromium(III), synergistic effect

## Abstract

In this paper, the response surface methodology (RSM) was proposed for studying the synergistic extraction of chromium(III) ions by double-carrier supported liquid membrane (DCSLM) with organophosphorus carriers (D2EHPA/Cyanex272). At first, the optimization method of “one-factor-at-a-time” was adopted for determination of the best conditions for Cr(III) extraction by SLM with only one carrier (D2EHPA). The optimum/threshold D2EHPA concentration in the membrane phase increased linearly with initial concentration of Cr(III) ions in the feed phase. After the addition the second carrier (Cyanex272), the synergistic effect was observed. The largest percentage of extraction and the shorter time was obtained. The optimization of the synergistic extraction in DCSLM system by RSM using Box–Behnken design (BBD) for three variables (concentration and proportions of the carriers, initial concentration of Cr(III), and time of the process) was studied. The statistical model was verified with the analysis of variance (ANOVA) for the response surface quadratic model. The reduced quadratic model showed that the predicted values were in agreement with those obtained experimentally, as well as the fact that the concentrations and proportions of the carriers had a significant influence on the response. The developed model was considered to be verified and can be used to predict the optimal condition for the chromium ions extraction.

## 1. Introduction

Heavy metals (e.g., chromium) and their compounds are among the worst water contaminants. They accumulate in the sediments and are toxic for living organisms. The highest oxidation state of chromium is +6, the lowest is −2; the +3 and +6 states are most common in chromium compounds. It is still problematic to remove chromium from wastewater efficiently [1]. On the other hand, according to the European Commission Report of 2010 [2], chromium belongs to the group of “critical elements”. This means that it is characterized by limited resources and the lack of substitutes, while being essential for economic and industrial development. Considering the above factors, it is crucial to explore and develop methods for efficient separation of chromium from aqueous solutions. There are several methods such as reduction, chemical precipitation, adsorption, ion exchange, and membrane separation that are quite popular in separation of heavy metals; however, some hope is also seen in using liquid membranes, mainly immobilized—SLM (supported liquid membrane). In the SLM, the pores of the polymer matrix fill the membrane phase. Typically, the polymer support is made of polypropylene, polyethylene, Teflon, polyamide, etc. Due to the facilitated carrier transport, the liquid membrane is the most effective technique for the selective separation of metals from aqueous solutions. The most popular types of ion metal carriers are crown ethers; organophosphorus compounds; and primary, secondary, tertiary, and quaternary ammonium salts [3]. However, in recent years, liquid membrane systems with a mixture of two extractants have become very significant in the separation of the metal ions [4,5,6,7,8]. Addition of extractant/carrier mixture in appropriate proportion into the membrane phase has a synergistic and positive effect on improving both the rate and selectivity of the separation process. However, our previous works [1,4] and experiences of authors [5,8] show that the introduction of a second carrier into the liquid membrane causes an increase in the number of variable process parameters that have a decisive impact on the efficiency of the process. It is necessary to select the most favorable proportions of both carriers and determine their correlations with the most important process parameters such as the initial concentration of transported ions, duration of the process, and membrane stability.

When many factors and interactions affect desired results, response surface methodology (RSM) is an effective tool for optimizing the process [9,10,11]. RSM, as a useful statistical and mathematical tool that is always used to develop, improve, and optimize the experimental process, which is affected by several factors. Compared with “one factor at a time” method, RSM cuts down material expense and time remarkably. In this methodology, analysis of variance (ANOVA) provides the statistical results and diagnostic checking tests that enable researchers to evaluate adequacy of the established models [11,12]. Hence, RSM not only provides the optimum level for each variable but also estimates interactions among them and their impact on one or more measured responses. [9].

Some authors [13,14,15,16] have already made successful attempts to apply this method in optimizing the metal ion separation in a liquid membrane system. Wongkaew et al. [13] studied the RSM for the separation of platinum (IV) across hollow fiber supported liquid membrane with different commercial extractants. RSM was used to qualify and estimate the influence of operating conditions (the concentration of the Pt (IV) in the feed phase and concentration of the carrier in the membrane phase) on the extraction efficiency. The predicted results were in good agreement with experimental data at a standard deviation of 1%. Liu et al. [14] used the RSM to optimization of vanadium (IV) extraction from stone coal leaching solution by the emulsion liquid membrane. All of the results revealed that RSM was successfully used to optimize the extraction process. Mesli and Belkhouche [15] also showed that the predicted values in lead recovery by liquid membrane were in good agreement with those found experimentally. Additionally, the authors proved that the carrier concentration has a significant individual effect on the response. Mondal and Saha [16] analyzed separation of hexavalent chromium from industrial effluent through the liquid membrane using the RSM. The authors found that the strip phase concentration, pH, and carrier concentration had the greatest influence on the transport of Cr(VI) ions. The comparison of experimental and predicted data by the RSM was then shown to be in good agreement.

In recent years, the results of research on the optimization of the membrane separation process using RSM have been published increasingly more often [5,6,7,13,14,15,16]. Therefore, it also seems promising to improve the extraction of chromium(III) by the RSM in the supported liquid membrane system. 

This work is devoted to the optimization of the chromium(III) separation process using experimental and statistical studies. The study presents the influence of the main process parameters (i.e., the concentration of carrier or carrier mixture in the membrane phase and the initial concentration of chromium in the aqueous phase) on the possibility of separation of Cr(III) from acid solutions with a double carrier supported liquid membrane. The research was carried out using the most commonly used organophosphorus carriers of the Cr(III) ions: bis(2-ethylhexyl) phosphoric acid (D2EHPA) and bis(2,4,4-trimethyl) phosphine acid (Cyanex272) [1,4].

The optimization method of “one experimental parameter at a time” was adopted for determination of the best conditions of Cr(III) recovery, from acidic aqueous solution by SLM with only one carrier (D2EHPA). In the next step, the modelling of the process was achieved by response surface methodology (RSM) using Box–Behnken design (BBD). BBD was used because, as reported by other authors [5,12], it is a method created for estimating a quadratic model and requires only three levels for each factor and specific positioning of design points, providing strong coefficient estimates near the center of the design space. This means it is significantly easier than other DoE methods, as less time is required, and no runs are include factors outside the min/max values of the studied area. For BBD, three parameters, namely, concentration and proportions of both carriers (D2EHPA/Cyanex272), initial concentration of Cr(III), and time of the process, were considered as factors of the quadratic model to predict the optimal extraction of Cr(III). 

## 2. Materials and Methods

### 2.1. Materials

Bis(2,4,4-trimethylpentyl) phosphinic acid (Cyanex272) was purchased from Cytec, Canada. Bis(2-ethylhexyl) phosphoric acid (D2EHPA) was purchased from Merck, Germany. Both substances were used to as an ions carrier. Chromium(III) chloride hexahydrate (CrCl_3_ 6H_2_O, ≥96% purity), hydrochloric acid (HCl), kerosene, and 1.5-diphenylcarbazide were obtained from Sigma-Aldrich, Poland. O-ksylene was purchased from Fluka, Poland. All chemicals were of analytical and reagent grade and were used as received without further purification. All aqueous solutions were prepared using deionized water. The immobilized liquid membrane was a PTFE porous polymer film with a pore size of 0.45 mm and porosity of 64% obtained from Sartorius, Germany.

### 2.2. Experimental Equipment and Procedure for SLM/DCSLM Transport Experiments

A detailed diagram of the experimental laboratory-scale unit has been reported elsewhere [1]. The separation equipment was composed of the vessel with two cylindrical chambers (capacity = 130 cm^3^ each) equipped with a mechanical stirrer each, and immersed in a water bath, pH meter (Schott, Germany), and thermostat with temperature and water level control system to ensure proper process temperature. Chambers were separated by membrane (active area 15.2 cm^2^) prepared by soaking PTFE porous polymer film for 24 h in a mixture of the carriers and organic solvents. The final concentration of the carriers in the membrane was changed in the range of 0–80 % *v*/*v*, while the volume concentration ratio of the kerosene/o-xylene was constant and equal to 2:1.

Feed solutions were prepared by dissolution the appropriate amount of the chromium(III) chloride in the deionized water. The final concentration was in the range of 0.025 to 1.000 g/dm^3^. As the stripping solution, 6 M HCl aqueous solution was used. The initial pH of the feed phase was equal to 4. The separation process was carried out at ambient temperature (T = 25 ± 0.5 °C) and atmospheric pressure. The samples were collected both from feed and receiving phase in the defined time intervals 

### 2.3. Determination of the Chromium(III) Ions Concentration

The spectrophotometric method was used for the Cr(III) ion concentration determination. Analysis was conducted using a NANOCOLOR UV/VIS NUV480 spectrophotometer (Macherey-Nagel, Germany) equipped with quartz cuvette cell, with path length of 1 cm. After solution intake, the sample was mineralized. The residue was diluted in deionized water, and stock solution of 1,5-diphenylcarbazide was added. The solution absorbance was measured at wavelength λ = 540 nm with a spectrophotometer. The concentration of the Cr(III) ions was read from the calibration curve. Each measurement was repeated three times. The Cr(III) ions concentrations are the average of the results obtained with the standard deviation value ± 0.0003.

### 2.4. Experimental Design for RSM

In accordance with previous studies [1,4], three factors, namely, concentration of the carriers in the membrane, initial concentration of feed phase, and time of the process, were chosen as the independent variables and designated as A, B, and C, respectively. The low, middle, and high levels of each variable (in coded forms −1, 0, and +1, respectively) are presented in Table 1 and Table 2. The statistical study was accomplished by Design-Expert 11 software. It was used for regression and graphical analysis of experimental data of Cr(III) extraction in SLM and DCSLM system. The number of experiments required to investigate this study was optimized by Box–Behnken design (BBD) at 16. Therefore, the experimental sequence was randomized to minimize the effects of the uncontrolled factors. In fact, the center point in the design was repeated three times for estimation of errors and curvature. The experiment points for BBD, shown in Table 1 and Table 2, were carried out in optimal conditions of Cr(III) extraction, found previously by the experimental study. The second-order polynomial was chosen as the BBD model to determine the regression equation that predicts the Cr(III) extraction.

## 3. Theory

The liquid membrane extraction process is most often studied using the one-factor-at-a-time (OFAT) method. In this method, one process variable is changed at a time, while others are kept on the same fixed level. This approach has several disadvantages, e.g., problems with the estimation of the interaction between process parameters may lead to overlooking optimal solution, and it is a time- and labor-consuming method. In recent years, design of experiments (DoE) and response surface methodology (RSM) are more willingly used to study, analyze, and optimize different industrial and laboratory processes [11,12].

The design of experiments (DoE) method is used for the selection of independent variables (process parameters) significantly affecting the process, development of the mathematical model fitting the obtained data, and the process optimization. The correct application of the method requires a multistep approach: defining the problem (process variables, responses, what we can measure, what we can optimize), strategy selection (selection of the variables or process optimization), design of the experimental matrix, conducting experiments, analysis of obtained data, development of the mathematical model and its evaluation, and model validation. 

The analysis of the obtained experimental results is carried out on normalized (coded) variables. Normalization of variables is the transformation of the independent variable real values into dimensionless values according to Equation (1).

(1) $$x_{i}=\frac{X_{i}-X_{i,0}}{\Delta X_{i}}$$

where *x_i_* is the coded value of the *i*-th independent variable, *X_i_* is the the real/uncoded value of the *i* independent variable, *X*_*i*,0_ is the real/uncoded value of the *i* independent variable at the center point, and Δ*X_i_* is the step change value of *X_i_* determined from Equation (2).

(2) $$\Delta X_{i}=\left(X_{i,}{}_{\max}-X_{i,}{}_{\min}\right)/2$$

where *X*_*i,*max_ is the maximum real value of the *i*-th independent variable, and *X*_*i,*min_ is the minimum real value of the *i* independent variable in the experimental space.

To find the optimal solution, it is necessary to use the polynomial function containing quadratic expressions. Most often, the second-degree polynomial equation is used (Equation (3)).

(3) $$Y=b_{0}+\sum_{i=1}^{k}b_{i}x_{i}+\sum_{1\leq i\leq j}^{k}b_{ij}x_{i}x_{j}+\sum_{i=1}^{k}b_{ii}x_{i}^{2}$$

where *Y* is the predicted response, *x_k_* is the coded value of independent variables, *k* is the number of independent variables used in the study, *b*_0_ is the constant, *b_i_* is the linear model coefficients, *b_ij_* is the linear model coefficients for interaction, and *b_ii_* is the quadratic model coefficients.

The obtained experimental results are used to calculate model coefficients. Then, the determination coefficients value are used to check the fit of the regression model to experimental results. The application of Fisher’s test allows for the determination of the individual model coefficients’ statistical significance. If the regression coefficient *b_i_* statistically significantly differ from 0, then the corresponding independent variables *x_i_* influence the response.

## 4. Results and Discussion

### 4.1. Subsection Influence of Carrier and Feed Concentration on the Cr(III) Ions Extraction in the SLM Method of “One Experimental Parameter at a Time”

**Figure 1:**
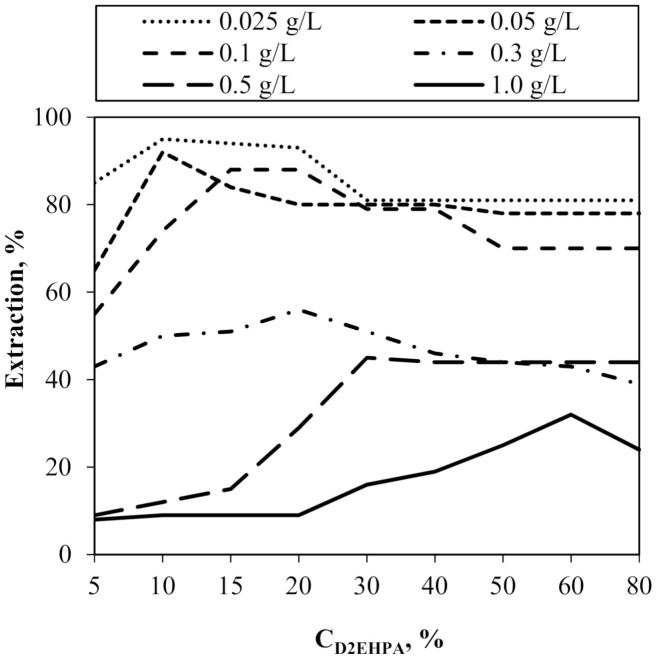
Extraction percentage depending on the D2EHPA concentration in membrane phase and Cr(III) ion concentration in the feed phase.

In the first step of the study, the optimization method of “one experimental parameter at a time”, was adopted to establish optimal conditions for the separation of the Cr(III) from acidic aqueous solution by SLM with D2EHPA carrier. To determine the effect of the concentration of the D2EHPA on the transport of Cr(III) ions in the studied system, we carried out the process for different initial concentrations of D2EHPA in the membrane and different initial concentrations of Cr(III) ions in the feed phase. The results of the experiments illustrated in Figure 1 confirm that D2EHPA had a high extraction ability for the Cr(III) ions. Carrier concentration in the membrane phase and initial concentrations of Cr(III) ions in the feed phase had a significant impact on the efficiency of the process. It is consistent with the experiences of other authors [17,18].

**Figure 2:**
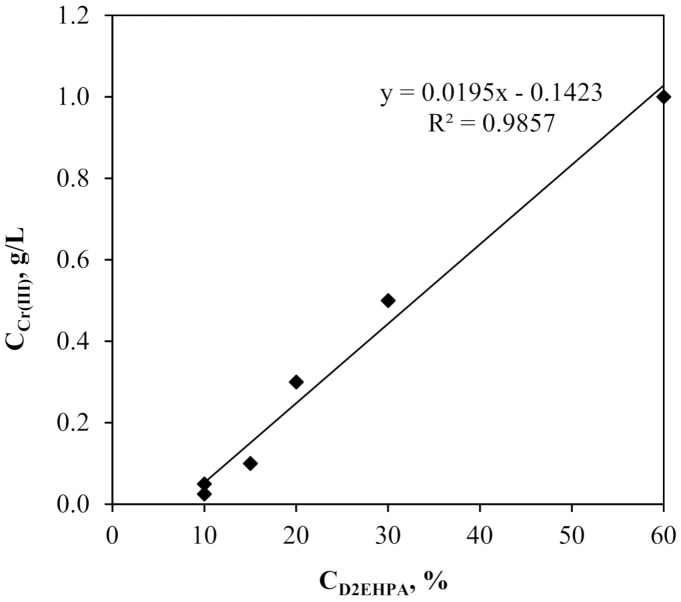
Relationship of D2EHPA threshold concentration in membrane phase to the initial Cr(III) ion concentration in feed phase.

It was observed that regardless of the initial Cr(III) concentration, there was a certain threshold concentration of the D2EHPA in SLM that guaranteed the highest efficiency of the process. The value of D2EHPA threshold concentration in SLM increased linearly with the initial concentration of Cr(III) ions in the feed phase (Figure 2, Table 3).

The time to reach the maximum extraction percentage (t_max_) was the shortest, and consequently the process rate was the fastest (Table 3). Moreover, results presented in Table 3 indicate that at higher initial concentrations of chromium in the feed phase, the extraction percentage was reduced. Achieving the maximum extraction percentage required a longer process time (t_max_). For example, for the initial concentration Cr(III) = 0.025 g/L, it was 0.5 h, while for 1.0 g/L, time extended to 10 h.

It can be concluded that the initial Cr(III) concentration is a limiting factor for the extraction efficiency in the SLM system. As reported by the authors [19,20], higher initial concentrations of Cr(III) cause high “exhaustion” of active carrier molecules at the interface surface (membrane phase/water phase). Moreover, the authors of [17] observed the increase in viscosity in the membrane phase, which also resulted in inhibition of the process. 

In previous works [1,4], we have shown that the modification of the SLM in the form of the addition of the second carrier to the membrane phase (Cyanex272) has a very beneficial effect on the Cr(III) ion transport efficiency. However, due to the need for a large number of long-term experiments, it is very difficult to carry out in a wide range of variable process parameters, such as the proportion of carrier concentrations and their dependence on the initial chromium concentration. In this work, we decided to use for this purpose the modeling of processes by response surface methodology (RSM) using Box–Behnken design (BBD) and three parameters, namely, concentration and proportions of both carriers (D2EHPA/Cyanex272), initial concentration of Cr(III), and time of the process (Table 1 and Table 2).

### 4.2. Effect of the Cyanex272 Addition to the SLM with D2EHPA RSM Modeling and Optimization of Chromium(III) Extraction (Step I)

BBD for the process variables of DCSLM is shown in Table 4, along with the experimental and the predicted responses. The first task was to find out which equation would allow for the obtaining of the best correlation between independent variables and responses. Analysis of variance (ANOVA) was carried out for most frequently applied equations: linear, two-factor interaction (2FI), quadratic, and cubic. On the basis of the achieved results, we found that the experimental data were described best with quadratic and linear equations. However, only for quadratic responses were high and satisfying values of R^2^ and adjusted R^2^ achieved. Therefore, the quadratic equation was selected for further analysis.

The statistical model was verified by employing ANOVA for the response surface quadratic model. As can be seen in Table 5, the Fischer variation (F-value) of the model was 6.13, indicating that the model is significant, but some terms of the equation were statistically not significant. The adjusted coefficient of 0.7548 was low where the predicted coefficient was negative, implying that the overall mean was a better predictor of the response than the current model. The difference between predicted R^2^ and adjusted R^2^ was larger than the recommended one (>0.2). This may demonstrate a large block effect or problems with models or data. The achievement of statistically significant value lack of fit (0.0497) is the incompliance of this model as this parameter should be statistically non-significant.

At a further stage of analysis, statistically non-significant terms of the initial equation were eliminated from the analysis. The reduction was made using a step-by-step method (from the most insignificant term). For both these response variables, only statistically significant terms were left, and higher R^2^, adjusted R^2^, and predicted R^2^ coefficients were achieved. Results of ANOVA are presented in Table 5. The regression reduced model equation for percentage Cr(III) extraction was expressed as Equation (4).

%E1 = 10.72 + 23.89A_c_ − 219.38B + 13.25C − 2.18A_c_^2^ + 159.38B^2^ (4)

where %E1 is the extraction percentage of Cr(III), A_c_ is Cyanex272 concentration, B is the initial Cr(III) concentration, and C is the time of the process. 

As seen in Table 6, the Fischer variation (F-value) of the model was 13.57, indicating that the model is significant. There was only a 0.03% chance that an F-value this large could occur due to noise. The lack of fit F-value of 5.36 implies that the lack of fit was not significant relative to the pure error. There was a 9.77% chance that a lack of fit F-value this large could occur due to noise. Non-significant lack of fit is desired because it is necessary to make the model best fit.

Predicted R^2^ shows the prediction of a response value estimated by the model. The difference between adjusted R^2^ and predicted R^2^ was expected to be in the range of 0–0.200 for the adequacy of the model. On this occasion, the predicted R^2^ of 0.6142 was in reasonable agreement with the adjusted R^2^ of 0.8073. Adequate precision is an estimation of the signal to noise ratio. A ratio greater than 4 is desirable, and hence the ratio of 13.069 implies an adequate signal. Therefore, this model can be used to navigate the design space.

**Figure 3:**
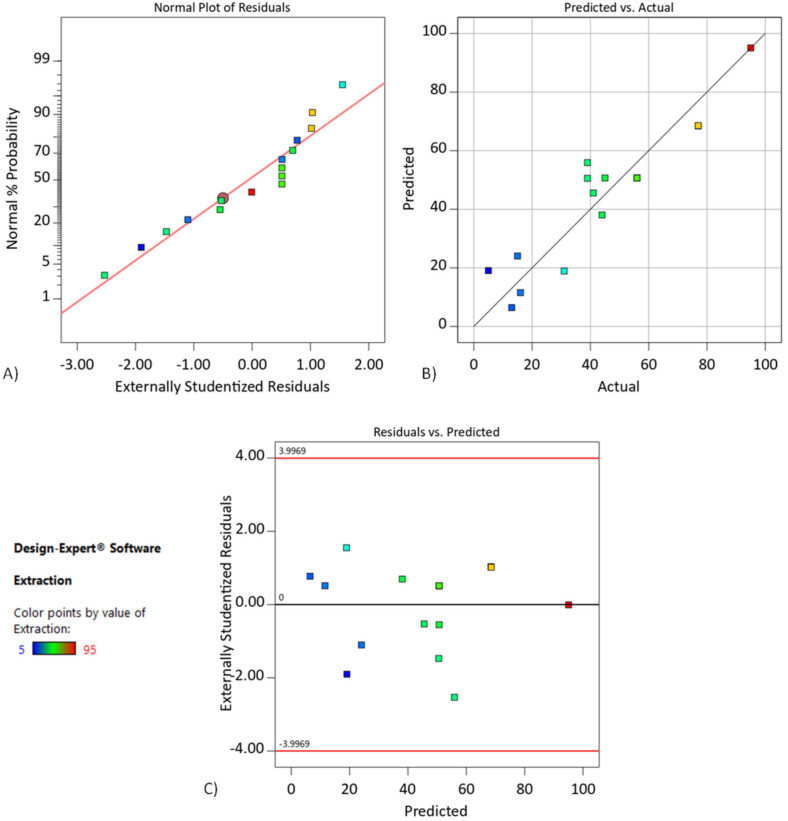
(**A**) The normal probability plot for chromium(III) extraction, (**B**) plot of observed values versus predicted values, and (**C**) plot of residuals versus observed values. DCSLM with threshold concentration of D2EHPA.

In Figure 3A, the normal probability plot of residuals for extraction of chromium(III) indicates how closely the set of obtained values followed the theoretical distribution. In Figure 3B, experimental points are reasonably aligned, suggesting a normal distribution. Thus, the developed regression model adequately describes the data obtained, approximately expressing 80.73% of the variability of response. Moreover, the use of residuals was investigated to access the model adequacy. In Figure 3C, residuals were found to be scattered and without any definite pattern, showing the adequacy of the model.

**Figure 4:**
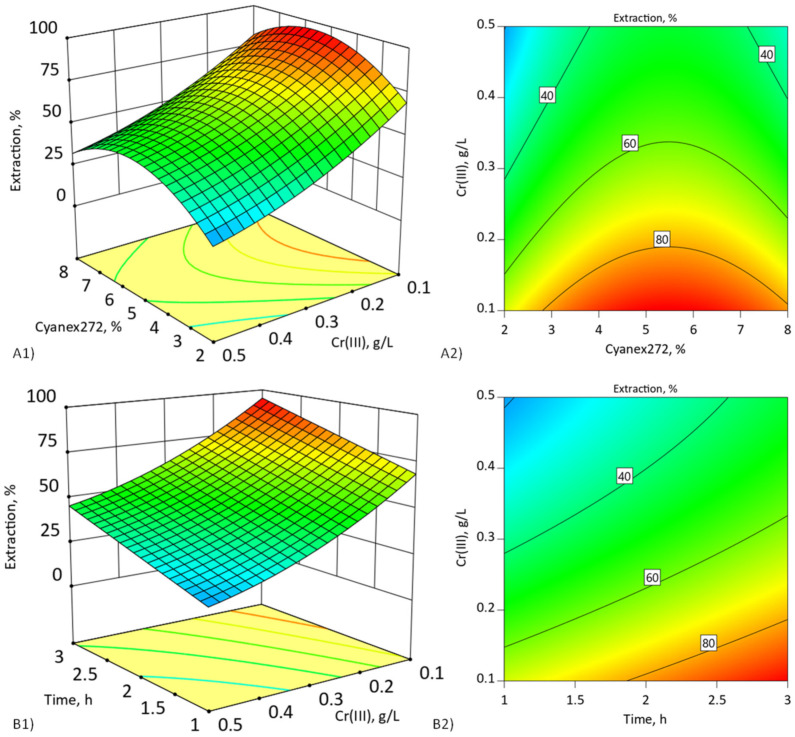
(**A1**,**A2**) Three-dimensional surface plot and contour plot of concentration of Cyanex272% (*v*/*v*) and chromium(III) (g/L) after 3 h extraction; (**B1**,**B2**) extraction time (h) and chromium(III) (g/L) for 5% *v*/*v* Cyanex272. DCSLM with threshold concentration of D2EHPA.

A Design Expert program was employed to plot the fitted response surface to understand the interaction of the most important parameters required for the optimum condition for the extraction of chromium(III). The plots are illustrated in Figure 4. According to Figure 4(A1,A2), the response surface plots showed the relation between the concentration of Cr(III) in the feed phase and the concentration of Cyanex272 in the double-carrier-supported liquid membrane, while the concentration of D2EHPA was kept constant at threshold concentration (selected and presented in Table 1).

The increase in the concentration of Cyanex272 (up to 5–6% *v*/*v*) improved percentages of extraction of chromium(III) because of the higher available Cyanex272 reacting with Cr(III) ions at the interface of feed phase [4]. The synergistic effect of both carriers was observed because the addition of 5% *v*/*v* Cyanex272 reduced the t_max_ of the Cr(III) ion pertraction to the membrane phase and improved the extraction percentage, regardless of the initial Cr(III) ion concentrations (Table 7) compared to membrane containing only D2EHPA (Table 3). However, at a higher Cyanex272 concentration (more than 6% *v*/*v*), the percentage of extraction decreased because of the limited mass transfer rate of Cr(III) ions across the liquid membrane phase. 

In the previous work [4], we proved that Cyanex272 dominates at the inter-phase surface in DCSLM system because of much higher surface activity than D2EHPA and is responsible for the complexation of the Cr(III) ions at the inter-phase surface on the side of the aqueous phase and transferring them to the transport structure formed by D2EHPA. The RSM modeling enables and facilitates the selection of the Cyanex272 threshold concentration in DCSLM system.

Figure 4(B1,B2) shows the interaction of the processing time with the initial concentration of chromium(III) and their relation to the percentage of extraction for selected Cyanex272 threshold concentration. It is typical for liquid membranes that maximum extraction is achieved for low concentration of transported substance [17]. At higher concentrations of transported substance, accumulation at the interface surface takes place, and results in fouling [20]. The fouling causes obstruction of the transportation of complex species across the liquid membrane phase, and extending the processing time will not increase the degree of extraction.

### 4.3. Effect of the D2EHPA Concentration in DCSLM with Threshold Concentration of Cyanex272 RSM Modeling (Step II)

Using the same methodology, we analyzed the effect of D2EHPA concentration on extraction with the Cyanex272 threshold concentration in DCSLM (Table 7). The aim was to determine the optimal proportions of both carriers in the DCSLM system. BBD for the process variables is given in Table 8, along with the experimental and the predicted response. The experiments also were verified using statistical analysis, and a modified quadratic model was proposed according to the results. The regression reduced model equation for percentage Cr(III) extraction was expressed as Equation (5).

%E2 = 20.06 + 4.34A_d_ − 305.00B + 17.25C + 3.75 BC − 0.07B^2^ + 281.25A_d_^2^ − 2.06C^2^ (5)

where %E2 is the extraction percentage of Cr(III), A_d_ is D2EHPA concentration, B is the initial Cr(III) concentration, and C is the time of the process.

The statistical model was verified by employing ANOVA for the response surface quadratic model. As can be seen in Table 9, the Fischer variation (F-value) of the model was 18.58, indicating that the model is significant. There is only a 0.02% chance that an F-value this large could occur due to noise. The lack of fit F-value of 5.79 implies that the lack of fit was not significant relative to the pure error. There was a 8.96% chance that a lack of fit F-value this large could have occurred due to noise. The predicted R^2^ of 0.7603 was in reasonable agreement with the adjusted R^2^ of 0.8913. The estimation of the signal to noise ratio was 14.844, and therefore this model can be used to navigate the design space.

**Figure 5:**
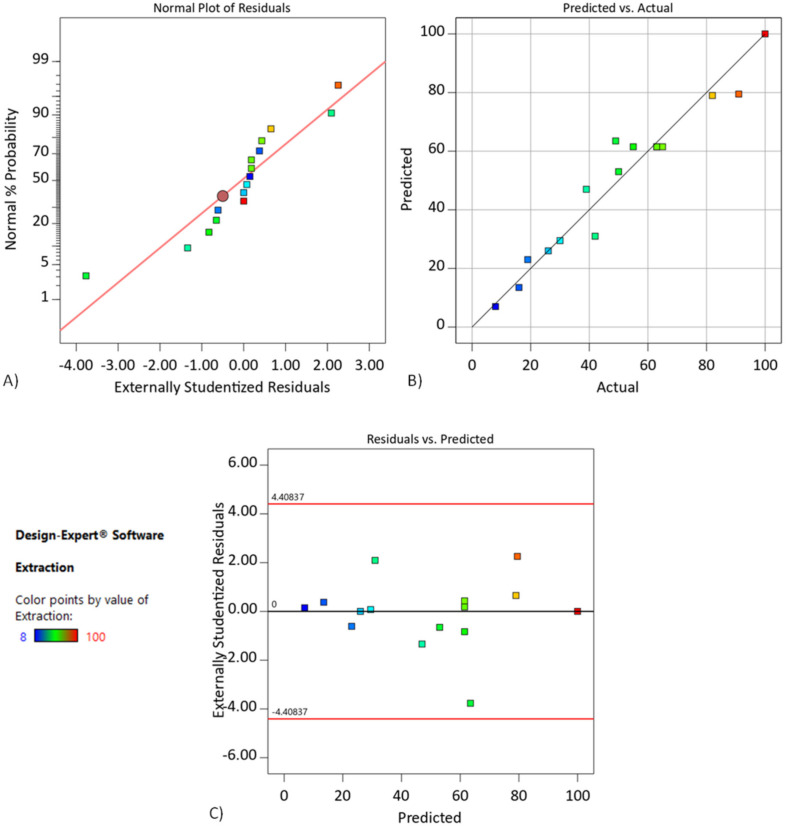
(**A**) The normal probability plot for chromium(III) extraction, (**B**) plot of observed values versus predicted values, and (**C**) plot of residuals versus observed values. DCSLM with threshold concentration of Cyanex272.

Figure 5A indicates how closely the set of obtained values followed the theoretical distribution. In Figure 5B, experimental points are reasonably aligned, suggesting a normal distribution. Thus, the developed regression model adequately describes the data obtained, approximately expressing 89.13% of the variability of response. As shown in Figure 5C, residuals were found to be scattered and without any definite pattern, which showed the adequacy of the model.

**Figure 6:**
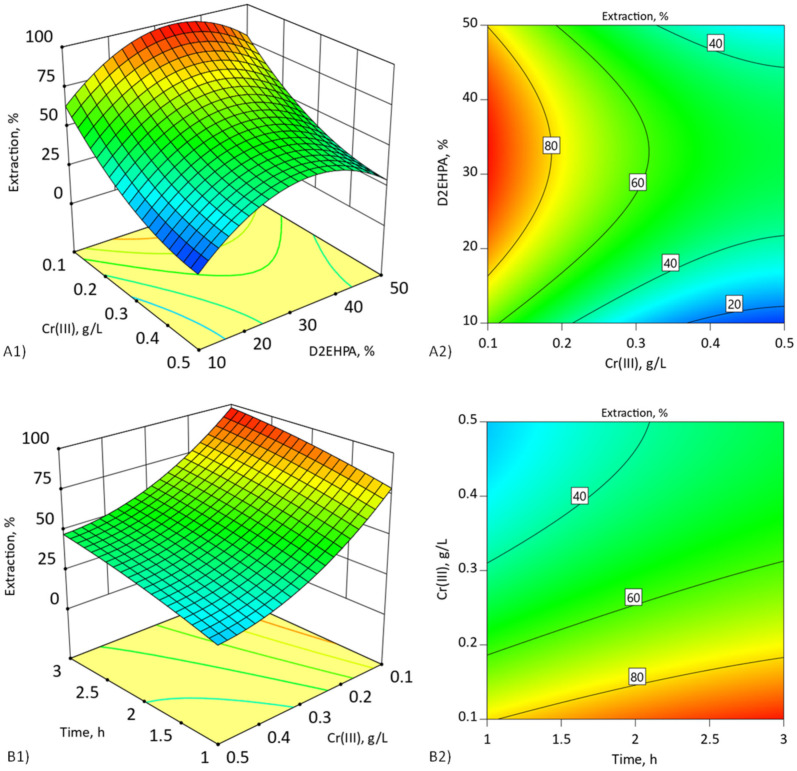
(**A1**,**A2**) Three-dimensional surface plot and contour plot of concentration of D2EHPA% (*v*/*v*) and chromium(III) (g/L) after 3 h. (**B1**,**B2**) Extraction time (h) and chromium(III) (g/L) for 30% *v*/*v* D2EHPA. DCSLM with threshold concentration of Cyanex272.

The 3D response surface plots and the contour plots are drawn visually to illustrate the main and interactive effects of the independent variables in Figure 6. According to Figure 6(A1,A2), the response surface plots show the relation between the concentration of Cr(III) in the feed phase and the concentration of D2EHPA in the double-carrier-supported liquid membrane, while the concentration of Cyanex272 was kept constant at threshold concentration.

The results show that the presence of two carriers in a suitable ratio (5% *v*/*v* Cyanex272 and 30–40% *v*/*v* D2EHPA) allows for the obtaining of the largest percentage of extraction, regardless of the initial concentration of Cr(III) ions. Moreover, the results summarized in Table 10 show that the selection of the optimal proportions of both carriers in DCSLM causes a significant increase in the percentage of extraction compared to a SLM with only D2EHPA. In the case of low initial concentration of Cr(III) ions (<0.1 g/L), shortening of time needed for complete extraction was also observed.

The addition of a second carrier (Cyanex272) threshold concentration to DCSLM allowed for an increase of 30% more of the concentration of D2EHPA in the membrane phase (Table 10). The presence of the Cyanex272 provides the possibility of better packing of the molecules of the D2EHPA and stabilizes the transport structure on the membrane phase [4]. Increasing the amount of carrier, which is responsible for the forming of a transport structure, creates favorable conditions for the rapid penetration of Cr(III) ions to the membrane phase and their efficient transport. 

**Figure 7:**
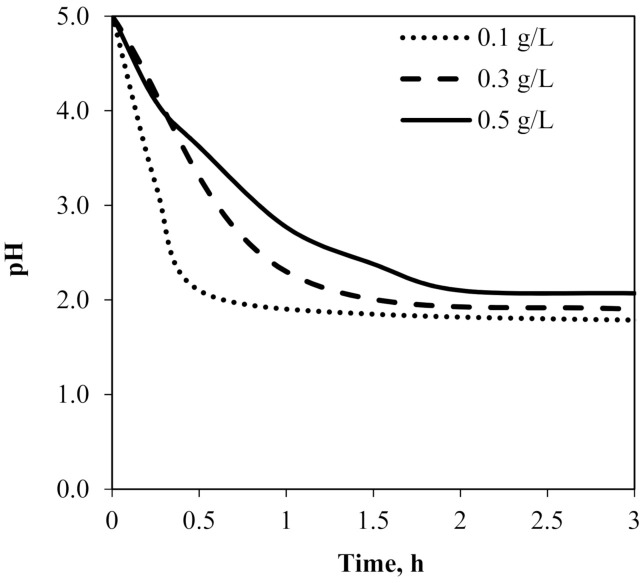
The pH changes in the feed phase during the Cr(III) ion extraction for optimum carrier proportion in DCSLM.

Figure 6(B1,B2) shows the interaction of the processing time with the initial concentration of chromium(III) and their relationship to the extraction percentage for selected threshold carrier concentrations (5% *v*/*v* Cyanex272/30% *v*/*v* D2EHPA). The maximum extraction of about 99% was observed for a low concentration of Cr(III) ions < 0.2 g/L after 3 h. For higher initial concentrations of Cr(III) ions > 0.3 g/L, the extraction percentage decreased up to 50 %. Analysis of the pH of the feed phase during the process with high initial Cr(III) ion concentration (>0.3 g/L) (Figure 7) showed that the high amount of accumulating Cr(III) ions in membrane phase and fouling effect can be caused by pH decrease—the driving force of the process [17,18]. It can be concluded that extending the duration of the process will increase the degree of extraction. 

The developed mathematical model was verified by conducting four additional experiments. The carrier ratio was set on a constant level of 5% *v*/*v* Cyanex272 and 20% *v*/*v* D2EHPA. The two other parameters (Cr(III) concentration and extraction time) were changed in the range of the experimental design limits. The experimental conditions, value of response predicted by model and experimental result, and statistical parameters (i.e., standard error, widths of the confidence, and tolerance intervals) are shown in Table 11. 

A satisfactory correlation was noticed between the experimental data and predicted values (R^2^ 87.17%). Thus, the developed model was considered to be verified and can be used to predict the optimal condition for the chromium ion extraction.

## 5. Conclusions

Experimental studies on separation of Cr(III) ions from aqueous solutions by the supported liquid membrane with D2EHPA carrier showed that the initial Cr(III) ion concentration is a limiting factor for the extraction efficiency. There is a threshold (optimal) concentration of the D2EHPA in SLM that guarantees the highest efficiency of the process. The value of D2EHPA threshold concentration increased linearly with increasing initial concentration of Cr(III) ions in the feed phase. Moreover, the synergistic effect was observed, which was characterized by an increasing in extraction efficiency after adding the second carrier (Cyanex272). 

The addition of threshold Cyanex272 concentration to SLM allowed for an increase of 30% more of the concentration of D2EHPA in the membrane phase. This increased the extraction percentage of the Cr(III) ions to the membrane phase and shortened the time of the maximum Cr(III) ion extraction to the membrane phase.

The presence of two carriers in DCSLM in a suitable ratio (5% *v*/*v* Cyanex272 and 30–40% *v*/*v* D2EHPA) allowed for the obtaining of the largest percentage of extraction, regardless of the initial concentration of Cr(III) ions compared to a SLM with only D2EHPA. In the case of low initial concentration of Cr(III) ions (<0.1 g/L), a reduction in the total extraction time was also noted.

The modelling of the process was achieved by response surface methodology (RSM) using Box–Behnken design (BBD). The DCSLM system has been successfully optimized. The experimental design, regression analysis, and quadratic models developed using response surface methodology according to BBD for the extraction efficiency were noticed to be reasonably accurate and effective in predicting the value of the response within the limits of the factors investigated. The reduced cubic of the quadratic model showed that the predicted values were in good agreement with those found experimentally, and the parameter of carrier concentrations/proportion had an important effect on the response.

A response surface methodology is an effective tool for optimizing the separation process using the supported liquid membrane technique. This tool greatly helps in carrying out research because it allows for the reduction in research time and minimization of its costs.

## Figures and Tables

**Table 1:** Level and code of variables for Box–Behnken design for DCSLM with threshold concentration of D2EHPA.

Varibles	Symbol	Coded Levels		
		−1	0	1
Cyanex272, % *v*/*v*	A_C_	2	5	8
Chromium(III), g/L	B	0.1	0.3	0.5
Time, h	C	1	2	3

**Table 2:** Level and code of variables for Box–Behnken design for DCSLM with threshold concentration of Cyanex272.

Varibles	Symbol	Coded Levels		
		−1	0	1
D2EHPA, % *v*/*v*	A_D_	10	30	50
Chromium(III), g/L	B	0.1	0.3	0.5
Time, h	C	1	2	3

**Table 3:** Selected D2EHPA threshold concentrations and time of the maximum Cr(III) ions pertraction in SLM for different initial concentrations of Cr(III) ions.

Initial Cr(III) Concentration, g/L	D2EHPA Threshold Concentration, %	t_max_, h	E, % (after 3 h)
0.025	10	0.5	95
0.050	10	0.5	92
0.100	15	1	88
0.300	20	3	56
0.500	30	9	45
1.000	60	10	32

**Table 4:** BBD design of process variables for experiment and values of experimental data for extraction of Cr(III) ions using DCSLM with threshold concentration of D2EHPA and different proportions of Cyanex272.

Experiment No.	Concentration of Cyanex272, %	Initial Concentration of the Cr(III), g/L	Time, h	Extraction, %	
	A_c_	B	C	Experimental	Predicted
1	−1	1	0	13	6
2	1	0	1	39	51
3	0	−1	−1	77	69
4	0	0	0	56	51
5	0	−1	0	77	68
6	0	1	0	31	19
7	−1	0	1	44	38
8	0	1	−1	5	19
9	0	0	0	45	51
10	1	0	−1	15	24
11	−1	0	−1	16	12
12	−1	−1	0	39	56
13	0	0	0	56	51
14	0	1	1	41	46
15	0	−1	1	95	95
16	0	0	0	56	51

**Table 5:** ANOVA for the response surface quadratic polynomial model for DCSLM with threshold concentration of D2EHPA.

Source	Sum of Squares	df	Mean Square	F-Value	*p*-Value	
**Model**	8610.69	9	956.74	6.13	0.0194	significant
**A**	312.50	1	312.50	2.00	0.2068	
**B**	4900.50	1	4900.50	31.41	0.0014	
**C**	1404.50	1	1404.50	9.00	0.0240	
**AB**	100.00	1	100.00	0.6409	0.4539	
**AC**	4.00	1	4.00	0.0256	0.8781	
**BC**	81.00	1	81.00	0.5191	0.4983	
**A^2^**	1540.56	1	1540.56	9.87	0.0200	
**B^2^**	162.56	1	162.56	1.04	0.3468	
**C^2^**	105.06	1	105.06	0.6733	0.4433	
**Residual**	936.25	6	156.04			
**Lack of fit**	845.50	3	281.83	9.32	0.0497	significant
**Pure error**	90.75	3	30.25			
**Cor total**	9546.94	15				

Std. deviation: 12.49; mean: 44.06; coefficient of variation %: 28.35; R^2^: 9019; adjusted R^2^: 0.7548; predicted R^2^: −0.4339; adequate precision: 8.101.

**Table 6:** ANOVA for the response surface reduced quadratic polynomial model for DCSLM with threshold concentration of D2EHPA.

Source	Sum of Squares	df	Mean Square	F-Value	*p*-Value	
**Model**	8320.63	5	1664.13	13.57	0.0003	significant
**A**	312.50	1	312.50	2.55	0.1415	
**B**	4900.50	1	4900.50	39.96	<0.0001	
**C**	1404.50	1	1404.50	11.45	0.0070	
**A^2^**	1540.56	1	1540.56	12.56	0.0053	
**B^2^**	162.56	1	162.56	1.33	0.2764	
**Residual**	1226.31	10	122.63			
**Lack of fit**	1135.56	7	162.22	5.36	0.0977	not significant
**Pure error**	90.75	3	30.25			
**Cor total**	9546.94	15				

Std. deviation: 11.07; mean: 44.06; voefficient of variation %: 25.13; R^2^: 8715; adjusted R^2^: 0.8073; predicted R^2^: 0.6142; adequate precision: 13.069.

**Table 7:** Selected Cyanex272 threshold concentrations and time of the maximum Cr(III) ion pertraction in DCSLM for different initial concentrations of Cr(III) ions. Based on the RSM analysis.

Initial Cr(III) Concentration, g/L	D2EHPA Threshold Concentration, %	Cyanex272 Threshold Concentration, %	t_max_, h	E, %
0.100	15	5	0.3	96
0.300	20		1	65
0.500	30		3	59

**Table 8:** BBD of process variables for experiment and values of experimental data for extraction of Cr(III) ions using DCSLM with threshold concentration of Cyanex272 and different proportions of D2EHPA.

Experiment No.	Concentration of D2EHPA, %	Initial Concentration of the Cr, g/L	Time, h	Extraction, %	
	A_d_	B	C	Experimental	Predicted
1	−1	1	1	16	14
2	0	0	0	63	62
3	−1	0	1	42	31
4	1	1	0	30	30
5	−1	0	−1	8	7
6	0	1	−1	26	26
7	0	1	1	50	53
8	1	−1	0	91	80
9	1	0	−1	19	23
10	1	0	1	39	47
11	0	0	0	65	62
12	0	−1	−1	82	79
13	−1	−1	0	49	64
14	0	0	0	63	62
15	0	−1	1	100	100
16	0	0	0	55	62

**Table 9:** ANOVA for the response surface reduced quadratic polynomial model for DCSLM with threshold concentration of Cyanex272.

Source	Sum of Squares	df	Mean Square	F-Value	*p*-Value	
**Model**	10,207.75	7	1458.25	18.58	0.0002	significant
**A**	512.00	1	512.00	6.52	0.0340	
**B**	3536.33	1	3536.33	45.05	0.0002	
**C**	1093.50	1	1093.50	13.93	0.0058	
**BC**	9.00	1	9.00	0.1146	0.7436	
**A^2^**	2756.25	1	2756.25	35.11	0.0004	
**B^2^**	506.25	1	506.25	6.45	0.0347	
**C^2^**	272.25	1	272.25	3.47	0.0996	
**Residual**	628.00	8	78.50			
**Lack of fit**	569.00	5	113.80	5.79	0.0896	not significant
**Pure error**	59.00	3	19.67			
**Cor total**	10,835.75	15				

Std. deviation: 8.86; mean: 49.88; coefficient of variation %: 17.76; R^2^: 9420; adjusted R^2^: 0.8913; predicted R^2^: 0.7603; adequate precision: 14.844.

**Table 10:** Effect of the optimum carrier proportion on the Cr(III) ions extraction in DCSLM compared to SLM. Concentration of the D2EHPA was selected on the basis of the RSM analysis.

Initial Cr(III) Concentration, g/L	Cyanex272 Threshold Concentration, %	D2EHPA Threshold Concentration, %	E, %	
			DCSLM	SLM/ (D2EHPA Threshold Concentration, %)
0.100	**5**	30	99 (after 2 h)	88/(15)
0.300		30	73	56/(20)
0.500		40	65	45/(30)

**Table 11:** The experimental conditions, value of response predicted by model and experimental results, and statistical parameters.

No. of Experiments	Cr, g/L	t, h	Response: E, %		Stdandard Deviation	SE Mean	95% CI Low for Mean	95% CI High for Mean	95% TI Low for 99% Pop	95% TI High for 99% Pop
			Predicted Mean	Experimant Results						
1	0.3	1.0	30.68	37	8,86	5.31	18.44	42.94	−18.24	79.61
2	0.3	2.0	42.88	54	8.86	4.14	33.32	52.43	−3.88	89.63
3	0.3	3.0	50.94	65	8.86	4.29	41.04	60.83	3.916	97.96
4	0.3	1.5	74.67	73	8.86	6.32	60.10	89.25	23.87	125.47

## Data Availability

Not applicable.
